# Performance of Zirconia for Dental Healthcare

**DOI:** 10.3390/ma3020863

**Published:** 2010-02-01

**Authors:** Nelson R.F.A. Silva, Irena Sailer, Yu Zhang, Paulo G. Coelho, Petra C. Guess, Anja Zembic, Ralf J. Kohal

**Affiliations:** 1 New York University, 345 East 24^th^ Street Room 804-S, New York, NY, 10010, USA; E-Mails: yz21@nyu.edu (Y.Z.); pgcoelho@gmail.com (P.G.C.); 2 University of Zurich, Plattenstrasse 11, 8032 Zurich, Switzerland; E-Mails: irena.sailer@zzmk.uzh.ch (I.S.); anja.zembic@zzmk.uzh.ch (A.Z.); 3 University of Freiburg, Hugstetter Straße 55, 79106 Freiburg, Germany; E-Mails: petra.guess@uniklinik-freiburg.de (P.C.G.); ralf.kohal@uniklinik-freiburg.de (R.J.K.)

**Keywords:** zirconia, oral implants, clinical performance, dental restorations, laboratory tests

## Abstract

The positive results of the performance of zirconia for orthopedics devices have led the dental community to explore possible esthetical and mechanical outcomes using this material. However, questions regarding long-term results have opened strong and controversial discussions regarding the utilization of zirconia as a substitute for alloys for restorations and implants. This narrative review presents the current knowledge on zirconia utilized for dental restorations, oral implant components, and zirconia oral implants, and also addresses laboratory tests and developments, clinical performance, and possible future trends of this material for dental healthcare.

## 1. Introduction

Considering all ceramic materials available for dental healthcare, zirconia offers so far the best mechanical properties. The good results obtained from orthopedic procedures brought significant confidence to dentistry for the utilization of zirconia as a support material (supposedly as a substitute for alloys) for esthetic restorations as well as for oral implants. However, controversies regarding to the proper interaction between the zirconia substrate and esthetic veneering porcelain arose, and in particular questions regarding veneered zirconia long-term performance for crowns and bridges. It is known however that significant improvements and developments of zirconia-ceramic systems combined with more controlled processing steps have broaden the future perspectives of this material. This narrative review addresses the current knowledge and future perspectives of the utilization of zirconia for dental healthcare emphasizing recent *in vivo* and *in vivo* findings. Some of data provided in this narrative review reflect 7–10 years of extensive studies developed on zirconia for dental applications by three recognized dental institutions that include University of Freiburg - Germany, University of Zurich - Switzerland and New York University - USA.

## 2. Zirconia as a Dental Material

Yttria-tetragonal zirconia polycrystals (Y-TZP) has been utilized for dental applications because of the extensively reported long-term success in the orthopedics field. However, in 2001 [1], several orthopedics components failed without a clear explanation, bringing up concerns regarding the long-term performance of such a material. After that, a significant number of articles investigating Y-TZP performance for medical and dental applications became available in the literature.

The flexural strength of zirconia rages from 800 to 1,000 MPa [2], which is very high compared to other dental ceramics. In addition to that, Y-TZP presents a stress-induced phase transformation mechanism that makes this material more resistant to crack propagation as a result of tetragonal to monoclinic (t-m) transformation which is accompanied by a volumetric expansion that closes crack tips and superimposes compressive stresses on the existing stress field [3]. High strength and fracture toughness explain why, when zirconia-supported restorations fail, the main failure mode resides in the veneering porcelain regardless of the processing method. Because of the importance of the porcelain failure issue from the clinical perspective, it will be discussed as a separate topic. As a counter part of the toughening mechanism, the t-m transformation also alters the phase integrity of the material and increases the susceptibility to the low-temperature degradation phenomenon [4]. The consequences of this aging mechanism are multiple and include Y-TZP grain pull-out and microcracking possibly leading to strength degradation [2]. Despite the conditions, which promote this aging process, the literature does not clearly address the relationship between low-temperature degradation and early failure events of the veneering porcelain. However, as the zirconia used in dentistry is identical to the ‘orthopedic’ grade zirconia, the issue of low temperature degradation must be kept in mind [5].

There are four zirconia-containing ceramic systems available for dental healthcare applications, which includes: Magnesium cation-doped partially stabilized zirconia (Mg-PSZ), zirconia-toughened alumina (ZTA), alumina-toughened zirconia (ATZ) and the most widely used yttrium cation-doped (3%) tetragonal zirconia polycrystal (3Y-TZP) [6]. The ATZ ceramic system has recently been used for fabrication of one-piece ceramic oral implants and will be discussed in the ceramic implant section including its clinical application.

Mg-PSZ has not been successfully used in dentistry because of the presence of porosities and large grain size (30–60 μm) that may lead to surface wear [7]. The Mg-PSZ processing method involves sintering temperatures between 1,680 and 1,800 °C which needs to be carefully controlled [6]. In addition, Mg-PSZ precursors free of SiO_2_ are difficult to obtain, or else they create magnesium silicates that can reduce the stabilizing Mg content and consequently lower the energy for the t-m transformation [8]. A dental ceramic system called Denzir–M (Dentronic AB, Skellefteå, Sweden) is an example of a fully sintered Mg-PSZ ceramic for dental crown and bridgework [9].

ZTA utilizes the stress-induced transformation capacity of zirconia in an alumina matrix [10,11]. One ceramic product available in the market, InCeram Zirconia (Vident^TM^, Brea, CA, USA), contains in one third (12 mol %) ceria-stabilized zirconia (12 Ce-TZP) added to In-Ceram^®^ Alumina^®^ [12]. This product can be processed via slip-casting or milling at a pre-sintering stage [2]. A positive aspect of the Ce-TZP ceramics is a better thermal stability and low temperature degradation response than Y-TZP ceramics under similar conditions [13]. As a drawback, ZTA shows a greater amount of porosity (between 8 to 11%) when compared to 3Y-TZP [14].

3Y-TZP (3 mol % Y_2_0_3_ as stabilizer) [7] has been utilized to manufacture orthopedic devices for more than 20 years [5]. In dentistry, it has been used for root canal posts since 1989, for orthodontic brackets since 1994, for implant abutments since 1995 and for all-ceramic restorations since 1998 [15].

The 3Y-TZP mechanical properties depend primarily on the grain size. Smaller grain sizes (<1 μm) are associated with lower t-m transformation rates [16]. However, the t-m transformation becomes minimal when the grain sizes are below approximately 0.2 μm, which reduces, on the other hand, the toughening mechanism [17]. The final sintering stage ranges from 1,350 to 1,550 °C. The sintering protocol may influence significantly the final grain size [18] and consequently the t-m stability of the 3Y-TZP [2].

### 2.1. Pre- and Fully-Sintered 3Y-TZP for Dental Application

**Figure 1:**
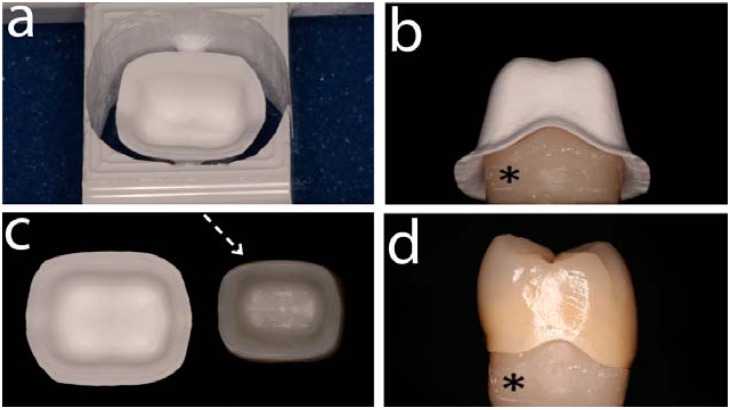
This sequence of images shows a molar crown restoration fabricated from a presintered zirconia (3Y-TZP) core (LAVA, 3M-ESPE). The pre-sintered 3Y-TZP block was machined (a) producing an enlarged zirconia framework core (b). Compare the core size related to scanned dowel (black asterisk). The pre-sintered milled block is then fully sintered obtaining the final 3Y-TZP core or framework for further porcelain veneer application. (c) shows the sintering shrinkage amounting to ~25% of the enlarged core as a compensation for the controlled sintering process. Note the shadow of the porcelain through the sintered core segmented (white arrow). (d) shows a mesial-side view of the final crown properly seated on the dowel (black asterisk).

The direct ceramic machining of pre-sintered 3Y-TZP became popular for the dental community in 2001 [19] and several manufacturers offer this product for all-ceramic restorations. The process starts from a die or wax pattern that is scanned by optical or contact scanners. Afterwards, a computer software (Computer Aided Design - CAD) designs an enlarged restoration and a pre-sintered ceramic block is milled by a milling system following the CAD (Figure 1). The pre-sintered milled dental framework is then fully sintered obtaining the final framework for further veneering porcelain application (Figure 1). The enlargement of the presintered framework is necessary due to the sintering shrinkage of approximately ~25%. Each company has its own milling and sintering method to obtain the final product. Some examples of pre-sintering systems are Cercon (Dentsply Friadent, Mannheim, Germany), LAVA (3M ESPE, Seefeld, Germany), Procera zirconia (Nobel Biocare; Gothenburg, Sweden) and IPS e.max ZirCAD (Ivoclar Vivadent, Schaan, Liechtenstein). The pre-sintering process reduces the chance of the t-m transformation and reduces the possibility of monoclinic phase surfaces. One advantage of this pre-sintering method is the possibility of using metal salts such as cerium, bismuth, iron or a combination of those to color either the pre-sintered 3Y-TZP blocks or the milled presintered frameworks. The process involves the immersion of the milled pre-sintered frameworks into 0.01 mol % of one (or a combination) of the salts [20]. This processing can create different shades of colors with a desirable esthetic effect for the final restoration. Another option of coloring the ceramics involves the addition of metal oxides to the starting ceramic powder [21].

If any subtractive procedure is performed after final sintering of the zirconia ceramic, for example sandblasting or grinding of the intaglio surface to increase the roughness for cementation purposes or adjustment of the same surface for better fit, a monoclinic phase will most likely be observed on the treated surface. This monoclinic transformation will in the first instance increase the strength of the restoration [22,23]. However, when a crack initiates in that area there is no transformation toughening mechanism to oppose crack propagation available anymore since the tetragonal phase was already transformed. The microcracks might extend through the developing monoclinic phase layer into the tetragonal phase area, which means that the monoclinic layer has no compressive impact on the crack [2,24]. Whether this treatment of the intaglio surface is of concern clinically long-term has to be shown. So far no clinically data on this topic can be found. Furthermore, grinding or sandblasting of surfaces with high (or mild/low) pressure ranges may or not lead to the formation of surface microcracking that could be detrimental to the long-term performance of the restorations, topic with conflicting statements in the dental literature.

Machining fully sintered 3Y-TZP blocks for dental restorations becomes a concern as this procedure has shown to create monoclinic zirconia [25] and surface microcracking which lower the long term expectation of the restorations [26]. In the absence of any clinical investigations reporting on effects of the aging phenomenon in dental applications, 3Y-TZP appears to have a strong potential as dental material as a result of its good mechanical properties. Although being aware of the possible aging phenomenon, but owing to improvements in monitoring the degradation, one should not expect a critical issue (e.g., framework fractures) with 3Y-TZP in the dental field [5]. However, several problems have been reported regarding the chipping or fracture of the veneering porcelain of all-ceramic restorations. This issue is not exclusive to a particular company or system and it may or may not be related with the t-m transformation at the veneer/core (framework) interface. The understanding and improvement of this chipping/fracture may dictate the future of zirconia for dental restorations.

## 3. Clinical Aspects of Zirconia Restorations

### 3.1. Clinical Survival and Complication Rates

All-ceramic materials are increasingly being used for the fabrication of crowns and fixed dental prostheses (FDPs). Due to their inferior load-bearing capacity compared to metals, however, ceramics have traditionally been applied in indications with lower loading forces.

Systematic reviews of the literature of all-ceramic and metal-ceramic reconstructions revealed that crowns made of ceramics exhibited survival rates similar to those of traditional metal-ceramic crowns [27]. Those crowns were made of reinforced ceramics such as glass-ceramics or glass-infiltrated alumina. The survival of all-ceramic FDPs made out of the same ceramics, however, was significantly lower than metal-ceramic FDP system [28]. Fracture of the ceramic framework was the main reason for failure of the FDPs [28]. Until today, based on these data metal frameworks veneered with tooth-colored ceramics still represent the standard for the choice of material for FDPs.

In contrast to other ceramics, zirconia has the potential to be applied as an alternative material to metal for reconstructions in indications with high loading forces, e.g., the posterior regions [29,30]. Currently, the data available on zirconia-based reconstructions confirm the load bearing capacity of this high-strength ceramic. Two studies are available reporting on the clinical performance of zirconia-based crowns at 2 and 3 years of follow-up, respectively [31,32]. The crowns were mainly located in the posterior regions on premolars and molars [31,32]. In one study no fracture of a crown was found [32], but in the other one molar crown fractured 1 month after cementation [31].

The six clinical studies published to date on zirconia-based FDPs showed promising results of the FDPs after observation periods of 3 to 5 years [28,33,34,35,36]. In these investigations, mainly posterior teeth were replaced. Low fracture rates of zirconia frameworks, ranging from 0% to 4.8%, were found [28,33,34,35,36]. Furthermore, fractures were reported in only two out of the six studies [28,37]. This result exceeds the findings of studies with FDPs made out of other ceramics. As an example, higher rates for fracture ranging from 10% [38] to 12% [39] were observed in two studies with posterior FDPs made out of glass-infiltrated ceramic after 5 years of clinical follow-up. The fracture rate of the “gold standard” metal-ceramic FDPs at 5 years amounts to 1.6% [28].

More recently, a randomized controlled clinical trial (RCT) comparing posterior FDPs with zirconia and metal frameworks has been performed [40]. At 3 years of follow-up no differences were found between the two types of FDPs. No fractures of ceramic or metal frameworks occurred, and the survival rate was 100% for both types of FDPs [40]. This result indicates that zirconia exhibits similar performance as framework material like metal. Hence, it may be concluded that zirconia performed better than the previous ceramics, however, the risk for fracture of the ceramic framework cannot be excluded [40]. Longer observation periods are needed before final conclusions can be drawn.

Yet, apart from excellent framework survival zirconia-based reconstructions were frequently subject to technical or biological problems [28,33,34,35,36,37]. The most frequently reported technical problem was chipping or fracture of the veneering ceramic [28,33,34,35,36,37]. This technical complication was reported in most of the investigations at incidences from 8% to 25% [28,35,36]. Compared to this, metal-ceramic FDPs have shown very low rates for chipping of metal veneering ceramic in the literature [41]. In the RCT of both types of reconstructions, no statistically significant difference of the outcome of the zirconia and metal veneering ceramics was found [40]. Still, the observations differed upon a clinically relevant level. While acceptable, minor chippings were found at a similar amount at both types of reconstructions (incidence: zirconia-based FDPs 25%, metal-ceramic FDPs 19.4%), extended defects of the veneering ceramic were only found at the zirconia-based FDPs (incidence: 8.4%). Those reconstructions were judged as clinically inacceptable and would need to be replaced in the daily practice [40].

**Figure 2:**
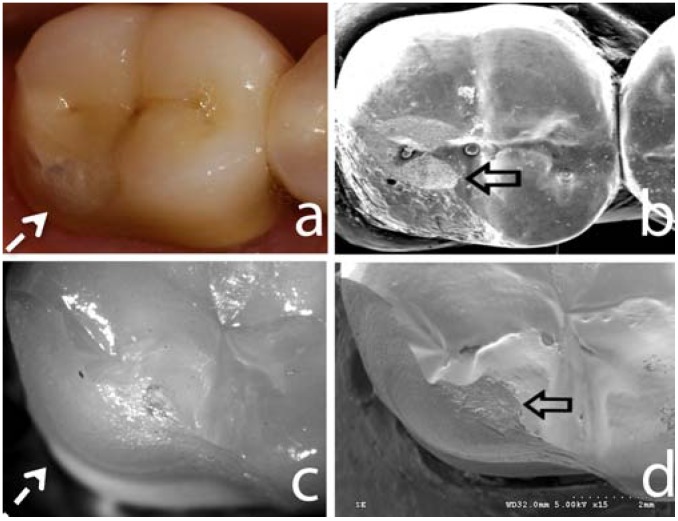
Image (a) shows a typical clinical fracture (segmented white arrow) of a zirconia-supported all-ceramic restoration after 3 years of service. Image (b) shows a SEM image of the correspondent fracture in (a). Open black arrow points to the rough surface created by occlusal adjustment, supposedly the fracture initiation site. Image (c) is a light polarized picture of fractured specimen obtained using the sliding contact fatigue testing method. Note the similar fracture pattern (d) that this method creates compared to the clinical fracture presented in (a), showing that this methods mimics the *in vivo* fracture modes. The SEM of the fractured site of the *in vivo* tested sample (d) is evidenced by the wear facet created by the indenter (open black arrow), similarly what is seen on the SEM image in (b).

Overall, zirconia veneering ceramics are one major concern for the clinical long-term outcome today. The reasons for the problems with the zirconia veneering ceramics still remain to be clarified. Several factors have been investigated in recent laboratory studies, which possibly affect the rate of veneering fractures. Among the factors analyzed are the thermal compatibility of the veneering ceramics and the zirconia frameworks [42,43], different surface treatments of the frameworks [44], the flexural strength of the veneering ceramics [44] and the bond strength between veneering ceramic and zirconia frameworks [44,45,46,47]. Final conclusions on the reasons cannot be drawn up to date. Yet, in the RCT, an interesting clinically relevant observation has been made [48]. It appeared that roughness of the veneering ceramic due to occlusal function or grinding was associated with the chippings (Figure 2). The analysis of the crack propagation direction revealed that the chippings in almost all FDPs had originated from a roughness of the ceramic at the occlusal region of the cusps [48]. This clinical finding is in accordance to recent laboratory investigations using a clinically relevant translational test design (Figure 2) and will be addressed herein as a separated topic. Hence, meticulous polishing of the rough surfaces resulting from grinding or occlusal function is crucial.

The main clinical factor to be considered at zirconia-based FDPs with respect to risk for framework fracture or chipping of the veneering ceramic is the design of the framework. On the one hand the design of the connectors of zirconia-based FDPs have to be adequate, on the other hand space for an even thickness of the veneering ceramic needs to be provided. In several studies computerized fabrication techniques by means of either CAM or CAD/CAM systems were used for the fabrication of the ceramic frameworks [28,33,34,35,36,37]. Some studies reported that special attention was paid towards adequate dimensions of the framework, or towards proper support of the veneering ceramic [28,35,36]. In the RCT for both types of FDPs the frameworks were manually modeled out of wax, respecting the anatomical situation of the patients and, hence, the support for the veneering ceramics was similar [40]. No framework fractures occurred, indicating sufficient clinical stability of the zirconia frameworks. Still, the zirconia-based FDPs exhibited more severe problems with the veneering ceramic [40]. Obviously, the support for the veneering ceramics cannot be considered a crucial factor for the greater extension of chippings at zirconia-based reconstructions.

Another observation made at the zirconia-based reconstructions was the occurrence of marginal discrepancies and marginal gaps, indicating problems with the fit of the computer-manufactured frameworks [28,31,32,33]. In one study, the problems with the fit were associated with a high occurrence of secondary caries, leading to the loss of the FDPs in some of the cases [28]. In this study, however, a prototype manufacturing procedure was used, and no comparison to the traditional metal-ceramic FDPs was done. When the two types of FDPs were compared like in the RCT, the marginal accuracy exhibited no differences from a statistical point of view [48]. However, clinically unacceptable marginal gaps were found only for two metal-ceramic FDPs, but occurred in six zirconia-ceramic FDPs [48]. It appears, that further refining the software of computer-aided systems is needed for an improvement of the accuracy in case of zirconia-based reconstructions.

Finally, the biological outcome of zirconia-based reconstructions generally was favorable in all studies [28,31,32,33,34,35,36,37]. Within the limitations of the observation period the promising survival rate of zirconia frameworks indicates this type of ceramic to be a valid alternative for metal frameworks. Higher rates of clinical complications, however, have to be taken into consideration. It is clear that longer observation periods are required in order to validate these medium term results.

## 4. Mechanical Response of Zirconia-Based Restorations

The understanding of the fracture and chipping mechanisms of zirconia-supported dental restorations became one of the most important steps in developing more predictable all-ceramic restorations. For that, fatigue testing performed on simplified and complex geometries specimens are necessary to firstly mimic oral mastication movements and secondly to follow the crack growth in 2D and 3D layered models. This section addresses the sample preparation and fatigue method employed at New York University College of Dentistry in an attempt to mimic *in vivo* fractures for ceramic systems with the main focus on zirconia-based restorations.

### 4.1. Flat Model Veneered Zirconia System

**Figure 3:**
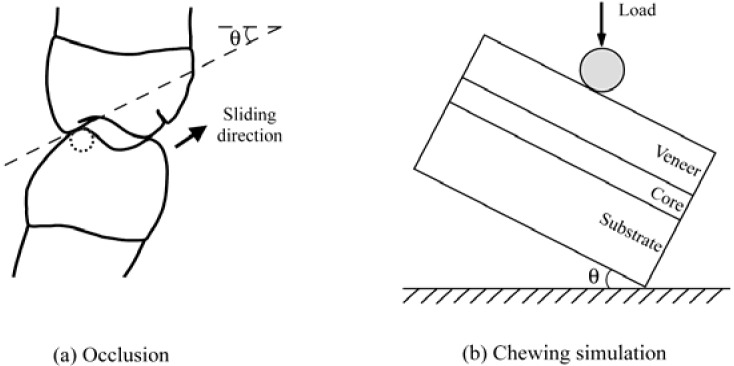
The schematic shows (a) posterior tooth contact in a chewing cycle as an eccentric contact of the mandibular buccal cusps with the inner inclines of the maxillary buccal cusps, followed by a sliding movement through centric occlusion, and then lifting off; (b) represents the sliding movement as a straight-line motion of a spherical indenter on the surface of a flat brittle layer (crown) supported by a complaint substrate (tooth dentin) with an inclination angle *θ* = 30°.

Occlusion is a complex phenomenon. However, as a first approximation, the posterior tooth contact in a chewing cycle can be visualized as an eccentric contact of the mandibular buccal cusps with the inner inclines of the maxillary buccal cusps, followed by a sliding movement through centric occlusion, and then lifting off (Figure 3a) [49]. The average length of the sliding path of a first molar is ~0.5 mm [50]. A typical inner incline of the maxillary buccal cusps has a radius of ~10 cm and a inclination angle of ~30° [50]. A usual diameter of the mandibular cusp tip is around several millimeters [51]. Since the sliding path is much shorter and the radius of the cusp tip is much smaller than the radius of the cusps inner incline, the sliding movement can be visualized as a straight-line motion of a spherical indenter on the surface of a flat brittle layer (crown) supported by a complaint substrate (tooth dentin) with an inclination angle *θ* = 30° (Figure 3b) [49,52]. Such a ball on flat layer system allows us to establish the essential damage modes occurring in veneered zirconia restorations [53].

Hertzian indentation fatigue tests have been performed on porcelain veneered zirconia structures on compliant substrates with spherical tungsten carbide indenters of radius *r* = 1.5 mm in water using a mouth-motion simulator (Elf 3300, EnduraTEC Division, Bose, Minnetonka, MN). Fully sintered CAD/CAM zirconia plates (15 mm in diameter and 0.5 mm thick, Lava^TM^ Frame, 3M ESPE, St. Paul, MN) were veneered with an overlay porcelain (1 mm thick, Lava^TM^ Ceram, 3M ESPE) by the manufacturer. The cementation surface of the zirconia core was roughened with 600-grit SiC abrasive paper and cemented (RelyX^TM^ ARC, 3M ESPE) to a composite block (15 mm in diameter and 4 mm thick, Z100^TM^, 3M ESPE), simulating veneered zirconia cemented to tooth-dentin structure. The porcelain surface was polished to 1 µm finish. The composite blocks were incubated in water for 4 weeks before cementation to allow for hydroscopic expansion. After cementation, the bonded structures were aged in water for 10 days before fatigue testing. The specimen was mounted on an inclined block (*θ* = 30°, Figure 3b). Load was applied in the vertical direction, but the loading consisted of a contact−load−slide−liftoff sequence: the indenter contacting the specimen, loading to a maximum while sliding down the surface to create a wear facet ~0.5 mm in length, unloading and lifting off from the specimen surface. A loading and unloading rate of 1,000 N/s was employed.

The current fatigue tests were designed to determine the type of damages and the number of cycles to failure *n*_F_ for a range of prescribed fatigue loads 120 N to 500 N. These load range covers the most part of the biting force range (35–485 N) observed clinically [54]. Following the fatigue loading, all specimens were subjected to post-mortem damage examination using combined optical microscopy (3D polarized specular reflection microscope, Edge R400, Micro Science Technologies, Marina Del Rey, CA) and a sectioning technique [55]. Failure of brittle layers on compliant substrates was defined when one of the occlusal surface crack systems reached the veneer/core interface or the cementation radial cracks popped-in. Damage maps were constructed for veneered zirconia on compliant substructures.

**Figure 4:**
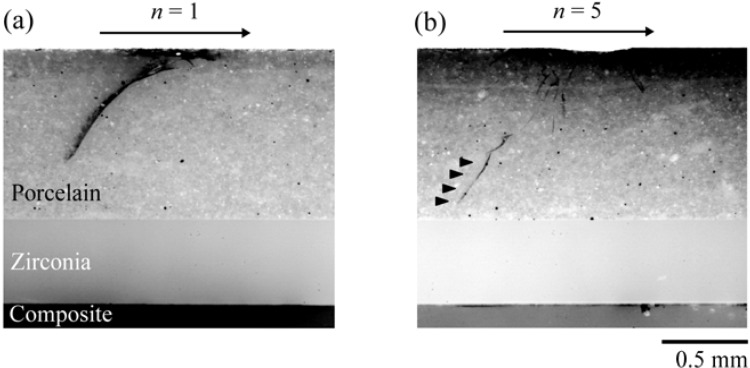
Figure 4 shows light polarized images of (a) a maximum fatigue load of 350 N. A partial cone crack has already propagated ~0.3 mm deep from the occlusal surface after the first loading cycle (black curvy line on the porcelain). (b) shows partial cones formed after 5 cycles. Black solid triangles point to the partial cone penetrating through the entire veneer porcelain.

**Figure 5:**
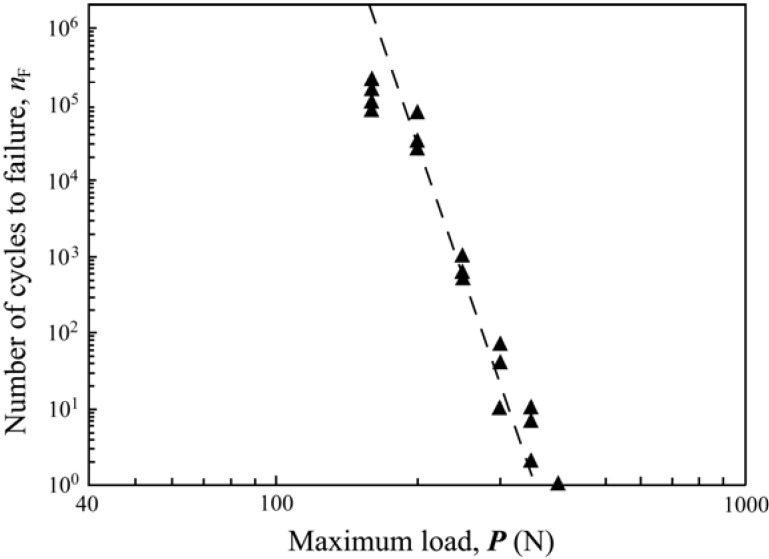
The graph represents a constructed damage map (load-cycles-type of failure) for porcelain/zirconia/composite trilayers subjected to off-axis fatigue loading .Note that for a high biting force (400 N or greater), cone cracks (black triangles) propagate through the entire porcelain veneers at first cycle. However, for a relatively low biting force (100–140 N), it takes several million cycles to propagate the cone cracks from the occlusal surface to the veneer/core interface.

For the range of loads tested here, a set of partial cones formed at the first contact−load−slide− liftoff cycle in porcelain veneers. Failure was predominately from the deep penetrating occlusal surface partial cone fracture. For example, for a maximum fatigue load of 350 N, a partial cone crack has already propagated ~0.3 mm deep from the occlusal surface after the first loading cycle (Figure 4a); it took only five cycles for the partial cones to penetrate through the entire porcelain veneer (Figure 4b). We then constructed the damage maps (load-cycles-type of failure) for porcelain/zirconia/composite trilayers subjected to off-axis fatigue loading (Figure 5). As can be seen, for a high biting force (400 N or greater), cone cracks propagate through the entire porcelain veneers at first cycle. However, for a relatively low biting force (100 – 140 N), it takes a couple million cycles to propagate the cone cracks from the occlusal surface to veneer/core interface, which is equivalent to 2 to 6 years *in vivo* [56].

In a veneered zirconia system, the stiff zirconia core provides stress shielding of the veneer layer and the underlying tooth structure. The exceptionally high strength of the zirconia core prevents flexure induced cementation surface radial fracture. The high modulus of the zirconia core (compared to porcelain) minimizes flexure of the porcelain veneer and propagation of partial cone cracks. Therefore, with the support of a stiff and strong zirconia core, flexure of the veneer layer is suppressed, resulting in a steady pace propagation of partial cones throughout the entire veneer layer. However, joining porcelain veneer with zirconia core at elevated temperatures poses another problem. Due to the difference in coefficient of thermal expansion and thermal conductivity between the porcelain veneer and zirconia core, residual stresses forms inevitable in the system upon cooling. These residual stresses can superimpose on the mechanical stresses induced from fatigue loading, resulting in premature fracture or chipping of the porcelain veneers. Giving the complex geometry nature of dental restorations, there is currently no effective method to tailor the residual stresses, and chipping or fracture of the porcelain veneer remains a major problem in veneered zirconia restorations.

### 4.2. Anatomically correct Zirconia-Supported Restorations

#### 4.2.1. Test Method

Various testing methods have been used to investigate the mechanical properties of dental materials. Many concerns have been raised lately regarding the clinical significance of simple traditional mechanical testing methodologies (single cycle loading/“crunch the crown test”) [57]. These tests reported unrealistically high fracture strength values largely overestimating the failure load. Furthermore the modes of failure were different than those reported in real clinical situations. In addition the results of these tests lack significant information considering the site of fracture initiation and fracture mechanics [54].

As described above, clinically, all-ceramic restorations commonly fail through stress corrosion and slow crack growth resulting from fatigue caused by repetitive occlusal contact [58].

This complex mechanical scenario of damage initiation and accumulation was reproduced in a recently developed in-vitro test design [59,60] by using mouth-motion fatigue loading of anatomically correct all-ceramic single crowns cemented onto a standardized mandibular molar model. Given that cyclic loading under wet conditions may reduce the initial strength of ceramics by 50% is pointing out that fatigue is a significant factor limiting the lifespan of all-ceramic restorations [61,62] that needs to be included in the test design.

The complex and patient unique geometry of posterior all-ceramic dental crowns represents a particularly interesting set of challenges to understanding stress concentration and damage evolution to fracture in response to loading.

#### 4.2.2. Failure Modes and Veneering Technique

Similar to flat model topic addressed previously, the sliding contact mouth-motion fatigue testing in water for anatomically correct crowns employed translation of a tungsten carbide indenter 0.5–0.7 mm along the crown surface from the disto-buccal cusp tip down toward the central fossa. Indenter translation simulates approximating tooth surfaces during mastication [59,60]. Failure progressed from the contact area through the body of the porcelain as noted in clinical cases [45], suggesting that the methodology employed was successful in simulating clinical scenarios (Figure 2).

During mouth-motion fatigue, various hand-layer veneered zirconia based crown systems resulted in a limited reliability - approximately 90% of specimens failed from veneer chip-off fracture by 100 K cycles at 200 N [60,63] reflecting the high failure rates within the veneering ceramic observed clinically.

Given the known highly accelerated failure from surface cracks with mouth-motion sliding contacts in water previously described, high failure rates within the veneers can be anticipated [49]. Fatigue properties of all-ceramic systems can also be related to flaw population (size number and distribution) inherent in the material from various fabrication processes as well as residual stresses. The sintering process for layering veneering ceramics is described as technique sensitive and subject to variability due to the individual building and multiple firing steps. However attempts to improve the microstructure and mechanical properties of veneering ceramics with development of glass-ceramic ingots for pressing veneering ceramics onto zirconia frameworks did not result in increased reliability [63,64]. Similar failure patterns with cohesive failure limited to the veneer material were observed [63,64]. Although high density of the veneering layer has been expected with the press technique, spherical porosities have been observed in the microstructure in the body of both layering and heat pressed veneers and also at the interface [63]. These porosities may be related to the fabrication processes as well as to the skill of the dental technician and may act as a stress raisers of low magnitude having little effect upon the fracture strength of the material [64]. Primary advantages of lost wax press systems are simplicity, efficiency and the ability to obtain improved marginal accuracy and to design anatomical characterization in defined thickness, of which the latter is difficult to achieve using the standard layering technique. A framework supported wax pattern can be tried in intraorally to allow adjustments in shape and occlusion prior to finalization of the press veneering process. Superior surface quality avoiding any corrections of the occlusal surface after firing might influence the performance of the press veneer positively. Promising results on zirconia frameworks veneered with an overpressed ceramic have been reported in so far only one published study on three unit posterior FPD. Sufficient strength with no chip fracture failure of the veneer has been observed within three years [37]. Major drawbacks of pressable materials are monochromatic colours with inferior aesthetic and optical qualities, which concern their application in the aesthetic zone of the mouth.

As the veneering ceramic material (flexural strength ~90–120 MPa) is weak compared to the high-strength core material (900 MPa) the veneering ceramic is prone to fail at low loads during the evolution of complex tensile fields in function. Thus all tested veneered Y-TZP crowns failed from cohesive fractures within the veneering ceramic, where a thin layer of veneering ceramic still remained on the zirconia coping [37]. As previously discussed in this review, this type of failure mode has also been reported clinically and indicates a sufficient interfacial bond between the core and the veneer material. The large chips observed for the Y-TZP veneer without exposure of the core/veneer interface strongly suggests high residual stresses within the veneer layer. This may be related to the very low thermal diffusivity of Y-TZP (~3 Wm/K) [65], which may affect the rate of cooling of the veneering porcelain. This cooling rate difference may lead to different stress states in the two systems [66]. Clinically, additional residual stresses may also result from place to place variation in thermal properties owing to irregular veneering ceramic thickness and the relative core veneer layer thickness ratio [67]. If these tensile stresses are not considered in the design of the restoration, failure can occur at unexpected low stresses [12].

The effect of the coefficient of thermal expansion (CTE) and the highly deleterious impact on core and veneering ceramics caused by residual stresses has been frequently discussed in the dental literature [43,68]. The pressable and layering veneering ceramic used in this research revealed CTE matching to that of the Y-TZP core and that may explain the absence of medial radial cracks and delaminations along the interface.

Catastrophic failure of the Y-TZP ceramic in terms of core cementation surface radial cracking was not evident in any of the veneered Y-TZP crowns and is consistent with most clinical observations [69,70]. The high crystalline content, flexural strength and fracture toughness of the Y-TZP based core material can be considered as reasons for the superior ability to resist subcritical crack propagation and stress corrosion in an aqueous environment [64].

#### 4.2.3. Framework Design

Framework design and crown geometry plays an important and underappreciated role in fracture failure of all-ceramic crowns [71]. Design practices of all-ceramic crown restorations have been based more upon empirical guidelines than upon clinically relevant scientific data. Remarkably little scientific data on optimal design of all-ceramic crowns or even of metal ceramic crowns has been published [72]. Whereas the overall crown thickness (minimum 1.5 mm recommended) may be of primary importance in resisting fracture [73], the relative layer thickness influences strength, stress distribution and failure mode. It has been suggested that a 1 to 1 ratio of core to veneering porcelain thickness may provide reasonable strength, esthetics and fabrication tolerance [73]. In an *in vivo* study it was stated that the fracture resistance increases as the core thickness/veneer thickness ratio increases [74].

With the beginning of CAD/CAM technologies in dentistry excessive veneer layer thickness were created due to uniform layer thickness of the copings for crowns as well as bar shaped connectors for FDPs. Ceramic copings are often milled to arbitrary thickness of 0.4 or 0.6 mm. Depending on tooth anatomy this may not provide uniform support or appropriate thickness for the veneering porcelain [75]. This problem was mainly caused by early software limitations. In the meantime, software improvements facilitate morphological adjustments of the framework to the later external contour of the restoration. A maximum layer thickness of the veneering material (2.0 mm [71]) can be defined. An additional scanning of a fully anatomic wax up of the restorations (double scan) was identified to be useful to accomplish the morphologic support by the framework.

Modern CAD/CAM systems are now able to provide a considerably better anatomically cut back framework design thus future clinical long-term results may be more favorable [36]. The amount of chip fractures within the veneering ceramic in studies with anatomically shaped framework design was very low (0% after 3 years [37] and 3.3% after 2 years [76] compared to results from other studies [75]).

In the above described standardized crown test model including mouth-motion-step-stress fatigue anatomical support of the veneering ceramic resulted in reduced chip size and significantly increased reliability with press and hand-layer veneering ceramics and appears to be a promising tool to optimize long-term performance of veneered Y-TZP crowns in clinical application (Guess, P. *et al.*, unpublished data; Silva, N.R.F.A. *et al.*, submitted data). Until more is known about clinical failure modes and clinical long-term performance parameters, precise recommendations cannot be made with confidence.

Further customization of milled zirconia copings to provide even and controlled porcelain thickness with the aim of decreasing cohesive porcelain fractures resulted in a modified framework design [72]. Appropriate porcelain and core thickness may decrease internal tensile stress, reduce mechanical failure, and optimize esthetics. The advantages of this coping design are controlled core and porcelain thicknesses and strength optimized marginal areas with a porcelain labial margin for esthetics and a high shoulder of zirconia and butt joints between the porcelain and core. Promising clinical results with no cohesive porcelain or core fracture have been encountered for the 150 customized milled zirconia crowns over a one year observation period. However, conclusions on the applicability of the described framework design cannot be drawn from such a small sample size and limited timeframe.

Moreover, increased dental laboratory technician time involved in full contour waxing, cut back and completing a second scan as well as the overall technique sensitivity of the design procedure and questionable feasibility for a general technician have to be considered with regard to this design proposal.

Nevertheless, the impact of framework design modifications on residual stress states needs to be addressed in further research. It should be stated that due to the low thermal conductivity of zirconia an evaluation of the existing firing programs for the veneering process of zirconia frameworks may be required to create a homogeneous heat distribution in bulky anatomically designed framework areas. Insufficient heat distribution may result in underfiring of the veneering ceramic, which may cause a higher amount of pores further eventually affecting the interface between the veneering and the framework ceramic [66].

#### 4.2.4. Innovative Veneering Techniques

Besides the high susceptibility to fracture, also from an economical point of view veneering of crown and FDP frameworks involving traditional methods such as the powder layering technique appears to be inefficient. An innovative veneering procedure for all-ceramic crown restorations using a CAD/CAM fabricated high strength lithium disilicate glass ceramic sintered onto a corresponding CAD/CAM fabricated zirconia coping has been described to be very promising with regard to initial mechanical strength testing [37]. Unpublished data (Guess *et al.*) using the above mentioned mouth motion fatigue test set up revealed no core or veneer failure at a load level of 900 N and 170 K cycles and confirmed the high reliability of this all-ceramic system.

Due to industrial prefabrication of the blocks and subsequent CAD/CAM processing a high quality material with a minimum of flaws compared to the manual procedures of veneering or heat pressing is provided. Given that appropriate software has been developed to ease the CAD phase for reconstruction the application for FPD frameworks would be of major interest as enhanced clinical performance can be expected. However CAD/CAM manufacturers have to provide the prerequisites regarding software and milling process accuracy before this technique can find general application. Long-term (5–10) year clinical evaluations on zirconia based crowns are however necessary to confirm the presented topics.

## 5. Zirconia Abutments for Oral Implants

**Figure 6:**
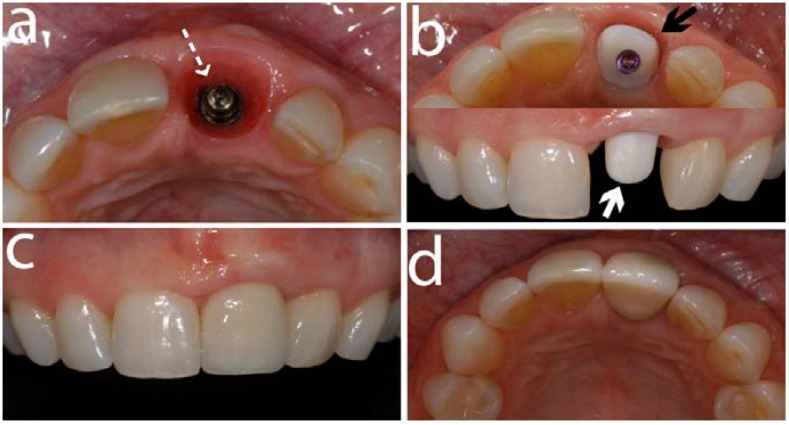
This sequence of clinical images shows a titanium implant (Straumann, Basel, Switzerland) placed (a) in a left central incisor area (segmented white arrow). (b) illustrates the occlusal view (black solid arrow) and front view (white solid arrow) of a zirconia abutment (Cares Abutment, Straumann, Basel, Switzerland) screw-retained on the implant. (c) and (d) are front and occlusal views respectively of the final restoration cemented on the abutment.

After the observations regarding the zirconia-based crowns and bridges we shift the focus to the behavior of zirconia as abutment material. The clinical applications of zirconia as abutment can be divided into two areas: 1) Pre- and custom-fabricated zirconia posts for endodontic treated roots. Although this procedure has been advocated by some dental clinicians, the use of single-visit fiber posts have proven to be the choice when an endodontic treated tooth needs to receive an endodontic post and built-up. 2) Zirconia abutments for oral implant reconstructions. Zirconia as implant abutment material (Figure 6) was first introduced in 1996 [77]. Based on its mechanical properties with the high fracture toughness compared to all other ceramics [78], zirconia seems not to be prone to fractures in clinical practice. In fact, until today there is no report on fractures of zirconia implant abutments in any clinical study [48]. However, long-term clinical trials on zirconia abutments are scarce and based on observation periods of three to six years [15,79,80,81]. Furthermore, most of the studies on zirconia abutments are case reports. For example, only four patients with six implants were treated in one of the above mentioned studies with the longest follow-up time of six years [80].

In order to be qualified for clinical use, ceramic abutments should perform like the “gold-standard“ metal abutments after being at least five years in function [82].

So far, only one randomized controlled clinical trial comparing zirconia and titanium abutments supported by 40 single implants was published [81]. After being in function for three years, 18 zirconia and 10 titanium abutments were followed-up. Both abutment materials exhibited survival rates of 100%, as well as similar biological and esthetical outcomes.

One specialty of zirconia is its crack resistance, also called “transformation toughening“ [7], previously addressed in this chapter. This phenomenon increases the fracture toughness of the material and could be the explanation for the so far excellent clinical survival rates of the abutments. On the contrary, *in vivo* studies demonstrated a decrease of 50% of the fracture toughness, when zirconia was exposed to a simulated 10-year aging process in a humid environment [83]. As there is no such clinical long-term data available, it remains unclear whether aging will reduce the physical properties of zirconia.

Based on the results of an *in vivo* study, zirconia abutments showed resistance to high loads of up to 738 N [84]. These loads exceed the ones occurring at implants in anterior regions, being in a range of 370 N [85].

The promising mechanical properties encouraged to apply zirconia abutments also in posterior regions, *i.e.*, mostly for the replacement of premolars [79,80,81]. The indication for zirconia abutments is thus not only limited to anterior regions, but is also justified in molar areas without failures [79,81]. Yet, small numbers of applied zirconia abutments in molar areas and the rather short observation periods have to be kept in mind.

### 5.1. Abutment Screw Loosening

Implant abutments are attached to oral implants via an abutment screw. Controversy regarding the clinical response of metal or zirconia screw retained implant abutments still remains. The incidence of abutment screw loosening does not differ significantly between metal and ceramic abutments [48]. An *in vivo* study defined the abutment screw to be the weakest component at titanium abutments, whereas it was not possible to identify the weakest component at zirconia abutments [86].

In a clinical study on zirconia abutments, 3.7% loosenings of the abutment screws were observed at four years [15]. This is in agreement with a similar study on titanium abutments, where screw loosening was found in 4% during a 5-year follow-up period [87].

No screw loosening occurred at externally connected zirconia abutments [81] and at internally connected zirconia abutments [79] at mean observation periods of 36 and 40 months. In both studies, customized abutments were fabricated by means of CAD/CAM procedures and screwed to the implants with a torque of 32 Ncm. The following factors may have contributed to these promising results:

1. The fit between implant and abutment is recommended to permit less than 5° of rotational movement in order to create a stable screw joint [88,89]. Tightening of implant components with a controlled torque of 20–30 Ncm reduces the risk of rotational movement and thus screw loosening [90,91].
2. Furthermore, an *in vivo* study on customized zirconia abutments fabricated by CAD/CAM procedures, exhibited an excellent fit with a rotational freedom of less than 3° [92].

When comparing the screw loosening rates at abutments with external and internal implant-abutment connections, a trend towards less problems was found at internally connected abutments in a systematic review [93]. A previous *in vivo* study analyzed the influence of the type of connection on the fracture load of zirconia abutments and found superior strength when there was an internal connection via a secondary metallic component [93]. Two-piece zirconia abutments thus are preferable in order to exhibit significantly higher bending moments compared to one-piece internally or externally connected abutments [93].

### 5.2. Fractures of the Veneering Ceramic

Fracture of veneering ceramic was addressed in a previous section but mainly on teeth-supported restorations. It becomes then imperative to discuss how all-ceramic restorations perform when the under layer support are zirconia abutments attached to titanium implants.

According to a systematic review on implant-supported single crowns, fracture of the veneering material was the third most common technical complication and appeared in 4.5% after five years [94].

No chipping occurred in one study at crowns supported by zirconia abutments, whereas chippings were found in 20% of crowns supported by titanium abutments [81]. Other studies reported chipping rates of 3.3% at 3 years and 8.3% at four years for single crowns supported by zirconia abutments [15,79].

Based on the promising experiences on zirconia abutments so far, a broadened indication area (for example a more frequent application in molar regions) can be expected in the future. More clinical studies with longer observation periods and increased numbers of observed zirconia abutments are definitively needed to draw final conclusions on the clinical long-term performance of zirconia implant abutments.

### 5.3. Biological Response on Mucosa and Bone

After the discussion on the mechanical aspects related to zirconia abutments, one could question the biological response to such material. In an animal study, it was shown that the collagen fiber orientation was similar around zirconia and titanium implant necks [95]. For both materials, the fibers run parallel-oblique and parallel to the implant surface [95]. Contrarily, around a natural tooth, collagen fibers of the periodontal ligament are oriented radially to the dental surface. The orientation of the collagen fibers is responsible for the formation of an adequate connective tissue seal and protection from plaque. The plaque protection from the peri-implant mucosa seems to be less effective due to this different collagen fiber organization around implants [96,97]. Both *in vivo* and *in vivo* studies showed less bacterial adhesion on zirconia compared to titanium surfaces [98,99,100].

In a clinical study, a similar degree of plaque accumulation was found at zirconia and titanium abutments at three years [81]. There was more plaque at natural teeth than at implant crowns supported by zirconia abutments [40,79]. Nonetheless, healthy soft tissue conditions were reported in all studies [15,40,79,81]. Less biological complications have been reported for ceramic abutments (5.2%) compared to titanium (7.7%), but the difference was not significant [48]

Regarding bone response when zirconia abutments are used as restoration support, there were no significant differences in bone levels between zirconia and titanium abutments after 3-year follow-up [81]. In addition, only minor bone level changes were observed until four years, being within the limits for successful implants [15,101]. In summary, there seems to be no negative effect of zirconia abutments on the peri-implant bone remodeling.

## 6. Zirconia Oral implants

In dental healthcare, the use of zirconia implants, as treatment option, is a new topic compared to the other dental applications described in this chapter. This implant system has been a subject of significant controversy among researchers and clinicians. Because of that, this section will review the current knowledge about such implants stating its *in vivo* performance and clinical expectations.

Oral implants are a clinically and scientifically proven method for the rehabilitation of edentulous areas in the oral cavity [102,103,104] and were introduced approximately 40 years ago [102,105,106,107,108,109,110].

Titanium has been the material of choice for the fabrication of oral implants, and many investigations have shown its long-term efficacy [111,112]. However, more recently, tooth-colored ceramic materials have been gaining popularity with dentists as well as with patients. The possible side effects of metallic materials [113,114] and the trend away from the use of metal in the human body have promoted the search for “more biocompatible” materials. As previously stated, zirconia has been used as an orthopedic implant material for many years and lately has been emerging in dentistry in the form of orthodontic brackets [115], post and core systems [116,117,118], all-ceramic prosthetic (FDPs) restorations [28,75], implant abutments [15,79], and more recently as a material for oral implants [119,120,121,122,123].

**Figure 7:**
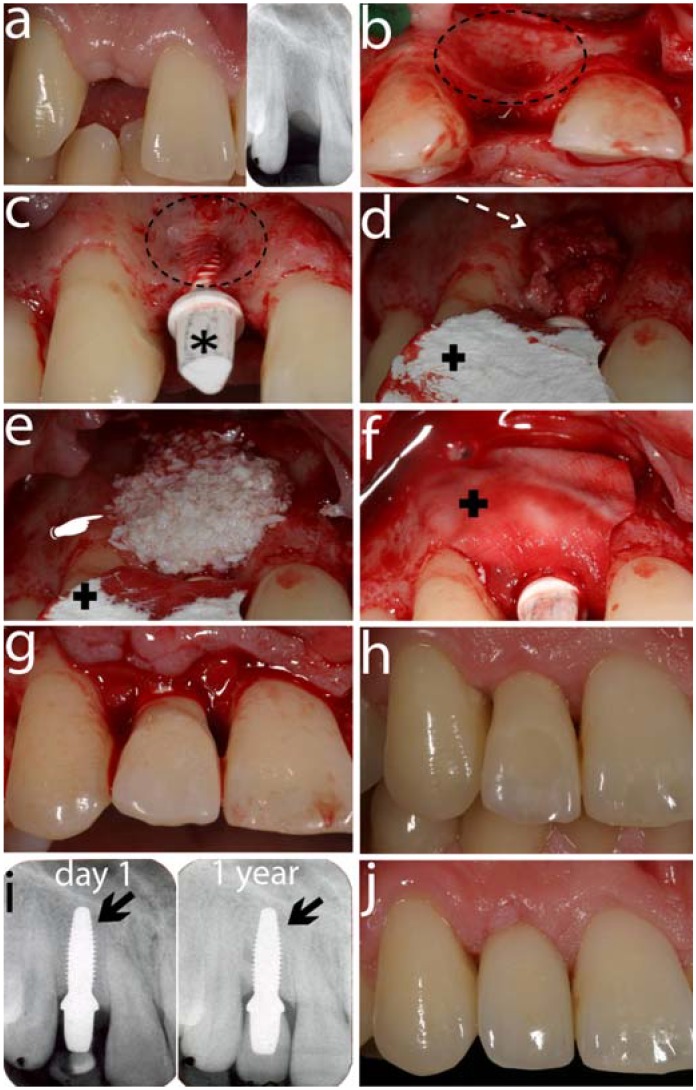
This figure shows a series of images of a clinical patient case treated with an alumina-toughened zirconia implant being placed in the area of a missing upper right lateral incisor.

The advantages of zirconia ceramic implants in comparison to titanium implants can be summarized in three points. Firstly, esthetics might be improved because zirconia implants are white and therefore present a better esthetic appeal than titanium (Figure 7). Secondly, as mentioned above, titanium is a metal and therefore potential health hazards may result from titanium particles and corrosive products provoking unwelcome host reactions. However, the clinical relevance of the findings is still not clear. Thirdly, if the number of remaining teeth decreases and implant-borne restorations are necessary, patients who request metal-free reconstructions only can be helped using ceramic implants.

(a) represents the clinical and radiographic situation. There is recognizable moderate to extended soft and hard tissue loss due to tooth loss. After opening of the surgical area (b), the soft tissue reflection reveals a pronounced bone deficiency in the area of former lateral incisor. The buccal bone wall was lost after tooth extraction (dotted-black circle). An alumina-toughened zirconia implant (Ziraldent, Metoxit, Thayngen, Switzerland) was placed in the area of the former lateral incisor area (c). The bone deficiency led to a dehiscence defect on the buccal aspect of the implant (dotted black circle. Note: proper positioning of the head of the implant (black asterisk) for further full crown preparation. The dehiscence defect (d) was treated with the guided bone regeneration concept using autogenous bone particles (segmented-white arrow) directly on the implants surface. A collagen membrane (BioGide, Biomaterials Geistlich, Wolhusen, Switzerland) was used in a later stage to cover the graft material (black cross). (e) The autogenous bone was covered subsequently with a bovine bone graft material (white pointer) (BioOss, Biomaterials Geistlich, Wolhusen, Switzerland) to prevent resorption of the underlying autogenous bone and to add volume to the area. After the graft material was in place covering the bone deficiency, the augmentation materials were covered with a porcine collagen membrane (black-cross) (BioGide, Biomaterials Geistlich, Wolhusen, Switzerland) to prevent dislocation (f). (g) After the augmentation procedure a provisional crown was cemented onto the implant. (h) shows the clinical situation after approximately 3 months of healing shows a healthy gingiva. Radiographs (i) at day of surgery (day 1) and after one year (1 year) show adequate osseointegration and position of the implant (black-solid arrows). (j) shows the 1-year clinical follow-up. The implant was restored with a lithium disilicate ceramic crown (IPS e.max CAD LT, IvoclarVivadent, Schaan, Liechtenstein).

Ceramic oral implants were introduced some decades ago. The material of choice at that time was aluminum oxide [110,124,125,126,127,128,129,130,131]. Alumina has a high hardness and high modulus of elasticity. Combined with moderate bending strength and fracture toughness it is prone to fracture. This could be the reason why there are no alumina implant systems remaining on the market. The material of choice for ceramic oral implants therefore seems to be 3Y-TZP. Compared to alumina, 3Y-TZP has a higher bending strength, a lower modulus of elasticity, and a higher fracture toughness. Laboratory investigations on the fracture strength of 3Y-TZP implants have shown that 3Y-TZP is able to withstand oral forces [132,133,134]. Furthermore, the results of animal experiments evaluating the osseointegration capacity of zirconia implants are promising. However, the clinical behavior of zirconia oral implants including bone remodeling and survival/success rates are of paramount interest as a basis for the clinical use of such ceramic implants in daily practice.

### 6.1. Laboratory Studies

A PubMed search yielded to laboratory investigations evaluating zirconia oral implants [132,133,134,135]. Three of the investigations evaluated the same type of one-piece zirconia implants [133,134,135]. One investigation evaluated the impact fracture resistance of a single-piece ceramic implant in comparison to two implant-titanium-abutment systems. The authors concluded that the fracture energy of the two titanium-abutment systems *versus* a single-piece Y-TZP implant in foam blocks was not different. However, the authors did not give any information on the fracture stability of the implants [135].

In a second investigation of the same group [133] data were presented on the reliability of as-received one-piece zirconia ceramic implants or after full crown preparation. The specimens were step-stress fatigued until failure or survival. The authors could not find a difference between the reliability of the two groups. They concluded that crown preparation did not influence the reliability of the one-piece zirconia ceramic implant and that fatigue did not influence the life-time of these implants at loads under 600 N. A different approach was chosen by a second research group [134] who evaluated the fracture strength of one-piece zirconia implants in a universal testing machine after artificial loading in the chewing simulator. Fifteen different groups (four titanium implant groups and 11 zirconia implant groups with different pretreatments) were investigated. Seven of the 72 artificially loaded implant samples failed in the chewing simulator. 3Y-TZP implant fracture occurred at 725–850 N when the implants were not prepared, and at 539–607 N when prepared. In contrast to Silva *et al.* [133], this study concluded that implant preparation had a significant negative influence on the implant fracture strength. However, the mean fracture strength values obtained by Andreiotelli & Kohal [134] obviously suffice oral loading for an extended period of time.

Two investigations reported on the fracture strength outcomes of prototype two-piece implants fabricated out of zirconia [123,132,136]. In the earlier investigation, the authors tested a special implant design (ReImplant^®^). Their results showed that the tested zirconia implant supporting all-ceramic Procera^®^ crowns may be able to withstand *in-vitro* physiological oral masticatory forces over a simulated 5-year-period [132]. In a later investigation, Kohal *et al.* [123,136] evaluated the fracture strength of two-piece cylindrical zirconia implants after aging in a chewing simulator. The values for the fracture strength after artificial loading with 1.2 million cycles for the two zirconia implant groups amounted to about 280 N. The biomechanical stability of the tested two-piece prototype implant groups seemed to be borderline for clinical use in respect to the average exerted occlusal forces. The zirconia implant groups showed fractures at relatively low fracture loads and therefore the authors cautioned the clinical use of these two-piece zirconia implants [123,136]. It is however surprising that no laboratory investigation of current commercially available zirconia implants systems is published in scientific journals so far.

**Table 1:** Animal studies reporting on bone-to-implant contact for zirconia implants.

Author (year)	Animal model	Bone-implant Contact
Akagawa *et al*. (1993) [137]	dogs	Unloaded implants: 82%
		Loaded implants: 70%
Akagawa *et al*. (1998) [138]	monkey	Loading period: 12 months
		Single freestanding implants: 54%−71%
		Connected freestanding implants: 58%−77%
		Implant-tooth supported: 70%−75%
		Loading period: 24 months
		Single freestanding implants: 66%−81%
		Connected freestanding implants: 66%−77%
		Implant-tooth supported: 66%−82%
Scarano *et al*. (2003) [139]	rabbit	4 weeks: 68%
Kohal *et al*. (2004) [151]	monkey	9 months; Y-TZP implants: 68%; Ti implants: 73%
Sennerby *et al*. (2005) [140]	rabbit	6 weeks
		Zr-Ctr: femur: 46%; tibia: 19%
		Zr-A: femur: 60%; tibia: 31%
		Zr-B: femur: 70%; tibia: 22%
		Ti-ox: femur: 68%; tibia: 24%
Hoffman *et al*. (2008) [141]	rabbit	2 weeks:
		Y-TZP: 55%
		Ti: 47.6%
		4 weeks:
		Y-TZP: 71.5%
		Ti: 80%
Depprich *et al*. (2008) [142]	minipig	1 week; Y-TZP: 35%; Ti: 48%
		4 weeks;Y-TZP: 45%;Ti: 99%
		12 weeks; Y-TZP: 71%; Ti: 83%
Lee *et al*. (2009) [143]	rabbit	3 weeks:
		ZiUnite: 70.5%
		Nano-modified surface A: 64.6%
		Nano-modified surface B: 62.2%
		TiUnite: 77.6%
		6 weeks:
		ZiUnite: 69.7%
		Nano-modified surface A: 68.6%
		Nano-modified surface B: 64.5%
		TiUnite: 67.1%
Kohal *et al*. (2009) [144]	rat	14 days:
		ZrO2modified: 45.3%
		TiUnite: 36.4%
		28 days:
		ZrO2modified: 59.4%
		TiUnite: 55.2%
Rocchietta *et al*. (2009) [145]	rabbit	3 weeks:
		ZiUnite: 27.5%
		Promimic: 42.5%
		CoAT sputtered: 36.1%
		TiUnite: 58.3%
Gahlert *et al*. (2009) [148]	minipig	4 weeks:
		Zirconia: 51.1%
		Ti-SLA: 55.1%
		8 weeks:
		Zirconia: 53,7%
		Ti-SLA: 70.4%
		12 weeks:
		Zirconia: 64.2%
		Ti-SLA: 54.4%

### 6.2. Animal Studies

Eleven animal studies reporting on osseointegration utilizing zirconia oral implants could be located (Table 1). Investigations evaluating only biomechanical stability (torque testing) or SEM analysis were not included. Akagawa and coworkers [137] evaluated bone-to-implant contact (BIC) of loaded and non-loaded zirconia implants placed in the lower jaws of dogs. There was a slightly higher BIC found for the nonloaded implants (82%) as compared to the loaded ones (70%). There was a loss of crestal bone evident around the loaded implants. A further investigation by the same group [138] revealed direct bone apposition (> 50% BIC) after two years in the different implant investigation groups. Scarano *et al.* [139] investigated the bone response of zirconia implants in the tibiae of rabbits. The BIC was reported to be 68%. In an investigation of loaded zirconia implants in an animal model, Kohal *et al.* [149] reported on soft and hard tissue integration. The mean height of the soft peri-implant tissue around zirconia and titanium implants was between 4.5 and 5 mm. No statistically significant differences were found in the extent of the different soft tissue compartments. The mean BIC after 9 months of healing amounted to 73% for the titanium implants and to 67% for the zirconia implants. The difference was not statistically significant. A comparison between different zirconia implant surfaces was performed by Sennerby *et al.* [140]. One particular modified zirconia implant surface showed a resistance to torque forces similar to that of oxidized titanium implants. The two tested modified zirconia implant surfaces showed a BIC in the femur of 60 to 70% (oxidized implants ~78%) and of approximately 20 to 30% in the tibia (oxidized implants ~25%). In a recent study, Hofmann *et al.* [141] compared bone apposition around four zirconia oral implants and four surface-modified titanium implants. The degree of BIC (zirconia two weeks: ~55%, titanium two weeks: ~47%; zirconia four weeks: ~71%, titanium four weeks: ~80%) was similar on all implants during an early healing phase. Depprich *et al.* [142] inserted acid-etched zirconia and titanium implants into the tibia of minipigs. Histological results regarding the bone-to-implant contact did not show statistically significant differences between the two groups at any timepoint (BIC: one week: zirconia 35%, titanium: 48%; four weeks: zirconia 45%, titanium: 99%; 12 weeks: zirconia 71%, titanium: 83%). Lee and coworkers [143] evaluated nano-technology-modified zirconia oral implants in rabbits. Three different zirconia implant groups (two with a nano-technology surface modification using calcium phosphate) were compared with titanium porous oxide implants. The titanium implants demonstrated a BIC of 77% after three weeks of healing and this BIC was significantly different to the nano-modified zirconia surfaces (BIC: 65% and 62% respectively). The non-nano modified zirconia surface had a BIC of 71% after three weeks. After a healing of six weeks the BIC values were 67% for titanium, 70% for the non-nano modified zirconia and 69% resp. 65% for the nano-modified zirconia surfaces with no statistically significant differences. In a second investigation by Kohal *et al.* [144], the authors presented the biomechanical and histological behavior of zirconia implants with no statistically significant different BIC values for rough titanium and zirconia surfaces. After 14 days of healing, rough titanium showed a BIC of 36% and rough zirconia of 45%. After 28 days the BIC amounted to 45% for titanium and 59% for zirconia. An investigation dealing again with chemically modified zirconia surfaces was performed by Rocchietta *et al.* [145] in rabbits. The authors investigated a topographically modified zirconia (this is similar to the non-nano modified zirconia surface investigated by Lee *et al.* [143] and the rough zirconia surface investigated by Kohal *et al.* [144] and furthermore two chemically modified zirconia surfaces (hydroxyapatite coating by dipping or sputtering). As controls oxidized titanium implants were used. The removal torque values for the different zirconia implants were not statistically significant different reaching from 29 Ncm (not HA coated) to 37 Ncm (sputter coated). The histological analysis revealed values for the BIC from 28% (not HA coated) to 58% (oxidized titanium) with no significant differences due to very broad 95% confidence intervals. The results are in agreement with those of Lee and coworkers [143] in that surface modifications using calcium phosphate coatings did not improve bone-to-implant contacts. In a series of investigations [146,147,148] biomechanical and histological results on zirconia and titanium oral implants were presented. Gahlert and coworkers [148] tested zirconia implants that were produced using a new low-pressure injection molding technique and surface treatment via acid-etching in the maxillae of pigs. Titanium implants with a sandblasted and acid-etched surface (SLA) served as control implants. The implants healed in for 4, 8, and 12 weeks. The BIC for the different implant groups and time points ranged from 27% to 51% for the zirconia implants and from 24% to 59% for the SLA-implants. No significant differences in bone-to-implant contact could be depicted.

### 6.3. Clinical Studies

Clinical investigations on zirconia oral implants are rare and of questionable scientific quality [120,121,149,150]. Three of these were retrospective observational cohort investigations [121,149,150]. Mellinghoff [150] evaluated in 71 patients 189 zirconia implants. The survival rate of the implants was 93% after one year. Of the 189 implants nine implants had to be removed and were counted as failure. Eight of these did not integrate during the healing phase. Only one implant fractured shortly after prosthetic reconstruction. In the second retrospective study presented by Oliva *et al.* [121] 100 zirconia oral implants in 36 patients were followed for one year. The investigated implant group comprised five different implant designs and two different surfaces. After one year the authors reported a success rate of 98% in the whole cohort. Over the investigated time period two implants (one of each surface) failed soon after implant installation showing implant mobility. In the last retrospective study, Lambrich *et al.* [149] compared titanium implants and zirconia implants. 361 implants (127 zirconia/234 titanium) in 124 patients were followed-up for an observation period of 21.4 months. The survival rate of the titanium implants amounted to 98% in the upper jaw and to 97% in the lower jaw: The zirconia implants showed a survival rate of 84% in the upper and 98% in the lower jaw. Eleven zirconia implants were lost, of these 10 implants in the maxilla and one implant in the mandible. The failures occurred in the healing period up to the first six months after loading. No fracture was mentioned as a reason for implant loss. None of the above-mentioned studies presented data on periimplant bone remodeling/loss.

The one year results of an ongoing prospective clinical investigation were presented recently in an abstract [123]. The objective of this 5-year, prospective, cohort investigation was to determine the success rate and bone remodeling after one year of a one-piece zirconia oral implant with a roughened surface. Ninety-two patients participated in the investigation. Patients were treated with one implant to replace a single missing tooth (n = 65) or two implants for a three-unit bridge (n = 27). In total 119 implants were placed. At the 1-year follow-up four implants were counted lost, resulting in a cumulative survival rate 96%. No implant fractures were observed. The average marginal bone loss occurring from implant installation to the 1-year follow-up amounted 1.6 mm. Almost a third (32%) of the implants lost 2 mm or more of bone.

### 6.4. Discussion and Critical Appraisal

#### 6.4.1. Zirconia Oral Implants and Stability

It seems that one-piece zirconia implants are stable enough to withstand normal occlusal forces. The ATZ implant systems (Figure 7) have been suggested to overcome the possible ageing phenomenon as previously described in this article.

The use of two-piece ceramic implants for intraoral application is of concern. The fact that there is no data available on the mechanical performance of current commercially available implants in the scientific literature is astonishing. Therefore, caution with their usage is highly recommended.

#### 6.4.2. Zirconia Oral Implants and Osseointegration

This section also discussed animal investigations using zirconia oral implants. All studies that compared zirconia with titanium implants show that the bone-to-implant contact was similar for both materials [140,141,142,143,144,145,148,151]. The above studies also revealed that bone grows similarly or even better onto zirconia as it grows onto titanium. Therefore zirconia can be recommended—from an osseointegration perspective—as material for the fabrication of oral implants.

#### 6.4.3. Clinical Investigations

It is noteworthy to mention that although several zirconia implant systems are available on the market (White Sky implant system from Bredent medical GmbH & Co KG; Ceraroot^®^ one-piece zirconia implant system from Ceraroot^®^; Sigma implants from Incermed SA; zit-z implants from Ziterion GmbH; Z-Look3 from Z-systems^®^) no valid clinical data is available in scientific journals. The methodology of the above-mentioned clinical studies is questionable. The only prospective clinical investigation [123] reported an increased bone loss around one-piece zirconia implants for a third of the inserted implants. Although zirconia was shown to integrate into bone comparable to titanium, currently, the scientific clinical data for zirconia implants is not sufficient to recommend zirconia implants for routine clinical use.

## 7. Future of Zirconia for Dental Healthcare

The problems involving early fracture of the veneer porcelain of zirconia supported restorations and the unclear effect of the low temperature degradation have led clinicians to question the total substitution of alloys through zirconia based dental restorations. As zirconia has demonstrated good mechanical and biological performance, future technology is attempting to improve esthetics and minimize veneer fracture, aiming to create confidence in the dental community towards this all-ceramic system. Few solutions for improving the zirconia-based dental work have been proposed and will be addressed in this section as possible alternatives to make zirconia dental structures adequate for long-term success during function in the patient’s mouth.

It is a challenge to comment on ‘aging free’ zirconia since the transformation occurring upon aging consists in a ‘natural’ return back to the monoclinic equilibrium state [1]. However, it is still not clear whether the t-m phenomenon compromises the final dental restoration. The addition of alumina to zirconia, creating zirconia/alumina composites has been advocated to reduce the aging kinetics maintaining good mechanical properties, and is being explored as one the possible options.

Robocasting technology generating 3D custom made layered structures is also being explored and shows to be promising for the future of zirconia for dental healthcare. “Robocasting is a freeform fabrication technique that utilizes computer-controlled extrusion of colloidal pastes, slurries, or inks” [152]. These machines can produce a variety of structures with changing or graded composition using a computer-aided design/manufacturing (CAD/CAM) program. The benefit of this technique is the possibility to design specific shapes/objects to be used for instance in bone defects surgeries. Transferring that to dental restorative applications, this technology would allow laboratories to produce dental restorations with different compositions of alumina and zirconia, therefore improving the mechanical characteristics of the final product.

Another possibility would be the creation of a functionally graded glass/zirconia/glass (G/Z/G) structure to overcome deficiencies of the veneered zirconia system [153]. This concept consists in coating the top and bottom of a pre-sintered Y-TZP with a slurry of glassy powder of SiO_2_-Al_2_O_3_-K_2_O-Na_2_O-CaO-Tb_4_O_7_. Hence the final product would be damage resistant, esthetically pleasing with increased translucency, and easy to cement, as the presence of glass would allow etching and silane application to enhance bonding mechanisms.

As it was shown in the present article, zirconia is being widely applied in dentistry starting from oral implant fabrication to the manufacturing of dental crown and bridgework. Some areas are however better investigated than others. Zirconia and zirconia-supported ceramics are worthy of being further evaluated particularly with improved production methods.
